# The Role of HE4 in the Follow-Up of Advanced Ovarian, Fallopian Tube, and Primary Peritoneal Cancer—CEEGOG OX-01 Study

**DOI:** 10.3390/cancers16213566

**Published:** 2024-10-23

**Authors:** Jiri Presl, Pavel Havelka, Vit Weinberger, Petra Ovesna, Peter Fekete, Filip Fruhauf, Marcin Jedryka, Branislav Bystricky, Aleksandra Strojna, Nataliya Volodko, Olga Matylevich, Petra Herboltova, Pawel Blecharz, Vladimir Kalist, Lucie Ehrlichova, Petr Stranik, Ladislav Masak, Renata Poncova, Andrzej Czekanski, Barbora Chaloupkova, Michaela Koblizkova, Vendula Smoligova, Marketa Hrabalova, Alena Jaksicova, Peter Linkesch, Libor Viktora, Jiri Bouda, Pavel Vlasak, Jan Kostun

**Affiliations:** 1 Department of Gynecology and Obstetrics, Faculty of Medicine in Pilsen, University Hospital Pilsen, Charles University, 323 00 Pilsen, Czech Republic; stranikp@fnplzen.cz (P.S.); smoligovav@fnplzen.cz (V.S.); boudaj@fnplzen.cz (J.B.); vlasakp@fnplzen.cz (P.V.); 2 Department of Gynecology and Obstetrics, KNTB a.s Zlin, 762 75 Zlin, Czech Republic; havelka@bnzlin.cz (P.H.); vladimir.kalist@bnzlin.cz (V.K.); barbora.chaloupkova@bnzlin.cz (B.C.); marketa.hrabalova@bnzlin.cz (M.H.); peter.linkesch@bnzlin.cz (P.L.); 3 Department of Obstetrics and Gynecology, University Hospital Brno and Medical Faculty, Masaryk University, 625 00 Brno, Czech Republic; weinberger.vit@fnbrno.cz (V.W.); koblizkova.michaela@fnbrno.cz (M.K.); viktora.libor@fnbrno.cz (L.V.); 4 Institute of Biostatistics and Analyses, Faculty of Medicine, Masaryk University, 625 00 Brno, Czech Republic; ovesna@iba.muni.cz; 5 Department of Gynecological Oncology, St. Elizabeth Cancer Institute, 811 08 Bratislava, Slovakia; peter.fekete@ousa.sk (P.F.); ladislav.masak@ousa.sk (L.M.); 6 Department of Obstetrics, Gynecology and Neonatology First Faculty of Medicine, Charles University and General University Hospital in Prague, 128 08 Prague, Czech Republic; filip.fruhauf@vfn.cz (F.F.); renata.poncova@vfn.cz (R.P.); 7 Gynecological Oncology Department, Lower Silesian Oncology, Pulmonology and Hematology Center, Wroclaw Medical University, 53-413 Wroclaw, Poland; mjedryka@interia.pl (M.J.); andrzej.czekanski@gmail.com (A.C.); 8 Department of Oncology, Faculty Hospital Trencin, Faculty of Healthcare, Alexander Dubcek University of Trencin, 911 06 Trencin, Slovakia; branislav.bystricky@fntn.sk; 9 Department of Gynecological Surgery and Gynecological Oncology of Adults and Adolescents, Pomeranian Medical University in Szczecin, 70-204 Szczecin, Poland; ola.strojna@yahoo.com; 10 Department of Gynecology and Gynecologic Oncology, Kliniken Essen-Mitte, 45136 Essen, Germany; 11 Department of Oncology and Radiology, Danylo Halytsky Lviv National Medical University, 79010 Lviv, Ukraine; nvolodko@yahoo.com; 12 Gynecologic Oncology Division, NN Alexandrov National Cancer Centre, 223040 Minsk, Belarus; omatylevich@tut.by; 13 Department of Gynecology and Obstetrics, Faculty of Medicine in Pilsen, Charles University, 306 05 Pilsen, Czech Republic; petraherb@email.cz; 14 Department of Gynecology and Obstetrics, Jihlava Hospital, 586 01 Jihlava, Czech Republic; 15 Department of Gynecologic Oncology, National Cancer Institute, M. Sklodowska-Curie Memorial Institute, 31-115 Cracow, Poland; pawel.blecharz@interia.pl; 16 Department of Internal Medicine-Hematology and Oncology, University Hospital Brno and Medical Faculty, Masaryk University, 625 00 Brno, Czech Republic; ehrlichova.lucie@fnbrno.cz; 17 Department of Oncology, KNTB a.s Zlin, 762 75 Zlin, Czech Republic; alena.jaksicova@bnzlin.cz

**Keywords:** ovarian cancer, tumor markers, CA125, HE4, recurrence detection

## Abstract

**Background**: Ovarian, fallopian tube, and primary peritoneal cancers often share clinical characteristics and are typically diagnosed at advanced stages due to nonspecific symptoms. The utility of tumor markers, particularly CA125 and HE4, in the diagnosis and follow-up of these cancers remains an area of active investigation. **Objectives**: The CEEGOG (Central and Eastern European Gynecologic Oncology Group) OX-01 study aimed to evaluate HE4’s role alongside CA125 in follow-up for advanced-stage ovarian, fallopian tube, and primary peritoneal cancers. It assessed the potential for detecting recurrence using marker elevation and imaging methods, examining the necessity of dynamic monitoring and current cut-off values’ accuracy for early relapse detection. **Methods**: In this multicenter prospective cohort study, 117 eligible patients with Stage III–IV cancers were included. Patients had elevated CA125 or HE4 at diagnosis and achieved complete remission after first-line treatment. HE4 and CA125 levels were monitored every 3–4 months in the first two years and every six months thereafter. CT scans were performed if markers exceeded set thresholds or increased by over 20%. **Results**: During a median follow-up of 13.7 months, 73% of patients relapsed. Median HE4 levels were significantly higher in relapsed patients. A 10 IU/mL increase from baseline in CA125 had a sensitivity of 83% and specificity of 93%, while a 15 pmol/L increase in HE4 had a sensitivity of 74% and specificity of 92% for predicting relapse up to three months before CT scan detection. **Conclusions**: The study found that dynamic changes in HE4 and CA125 levels, rather than predefined cut-off values, are crucial for early relapse detection. These markers may offer a significant lead time over imaging, potentially enabling earlier intervention. Further research is needed to validate these findings.

## Simple Summary

Ovarian, fallopian tube, and primary peritoneal cancers are often diagnosed at advanced stages due to nonspecific symptoms. This makes early detection challenging and impacts patient outcomes. This study sought to clarify the role of HE4 and CA125 in the follow-up of ovarian cancer patients and, potentially, improve early detection and management of recurrences. By monitoring changes in these markers over time, the researchers hoped to find a more reliable way to identify cancer recurrence before it is visible on CT scans. The findings suggest that tracking increases in these markers can predict a relapse earlier than CT scans, potentially allowing for earlier treatment. Further research is required to refine follow-up strategies and enhance outcomes for ovarian cancer patients.

## 1. Introduction and Background

The ovarian, fallopian tube, and primary peritoneal cancers are often grouped due to their similar embryological origins, pathological features, and clinical behavior [1]. Ovarian cancer is the most lethal gynecological cancer and has a reputation for being a “silent killer”. Indeed, due to its vague and nonspecific symptomatology, ovarian cancer tends to be diagnosed at an advanced stage. It is estimated that approximately 65,000 women will be diagnosed with, and 42,000 women will die from, ovarian cancer in Europe in 2024 [2].

The diagnosis of ovarian cancer remains a significant challenge. Currently, no effective screening method is available. Ultrasonography alone has proven insufficient, and tumor markers have shown limited reliability. Large epidemiological studies [3,4,5] on screening methods have revealed that adding a tumor marker to ultrasonography can help detect more early-stage cancers. However, this approach has not significantly impacted overall survival rates.

Ultrasound examination in the hands of an expert sonographer is crucial for distinguishing between benign and malignant adnexal disease. Various models based on ultrasound examination have been described and published; for example, IOTA (International Ovarian Tumor Analysis) simple rules and the ADNEX (Assessment of Different NEoplasias in the adneXa) model have very high sensitivity and specificity in ovarian cancer detection [6,7,8]. Although the tumor markers CA125 and HE4 (ROMA index—Risk of Ovarian Malignancy Algorithm) reveal inferiority in differentiating adnexal pathologies, their combination is still widely used. Both biomarkers are elevated in almost 80% of advanced epithelial ovarian cancer (EOC) patients [9]. HE4 has been shown to have higher specificity than CA125 for ovarian cancer diagnosis, particularly in premenopausal women and in patients with benign gynecological conditions [10].

The role of tumor markers in the follow-up of ovarian cancer patients varies widely. Several papers deal with this topic in the form of a review [11,12,13,14]. In some countries, there is no routine follow-up after treatment for ovarian cancer, and management is based solely on the emergence of symptoms. Both ESGO and ESMO recommendations state that there is no standard protocol or frequency for follow-up [15]. A reasonable approach involves assessing the patient every 3 to 4 months for the first two years and then every six months for 3 to 5 years. Follow-up can be individualized according to prognostic factors, and treatment modalities such as maintenance therapy and follow-up after five years should be discussed individually.

Our study investigated the possibility of detecting disease recurrence after first-line therapy during follow-up using CA125 and HE4 serum levels, clinical examination, and imaging methods when indicated. The study investigated whether a single tumor marker level measurement is sufficient or if the trends of marker levels over time are more significant in determining the recurrence, and evaluated the lead time to the increase of tumor marker levels before epithelial ovarian cancer recurrence was diagnosed by a CT scan. By addressing these questions, our study sought to clarify the role of HE4 in the follow-up of ovarian cancer patients and, potentially, improve early detection and management of recurrences.

## 2. Materials and Methodology

This was a multicenter prospective cohort study undertaken in 12 centers under the umbrella of CEEGOG (Central and Eastern European Gynecologic Oncology Group). Five centers were in the Czech Republic, two in Slovakia, three in Poland, and one each in Ukraine and Belarus. A standardized protocol and a bespoke online case report form (eCRF) were used for follow-up and data collection. Patients ≥18 years old diagnosed with Stage III–IV [16] high-grade serous, low-grade serous, endometroid, clear cell, undifferentiated invasive ovarian cancer, fallopian tube cancer, or peritoneal cancer were considered potentially eligible for inclusion. Moreover, it was required that they have an initial elevation of one or both tumor markers CA125/HE4 (CA125 > 35 IU/mL, HE4 > 140 pmol/L) on diagnosis followed by complete remission (CR) after first-line treatment based on CT abdomen + CT thorax (RECIST), negative CA125 (≤35 IU/mL), and negative HE4 (≤140 pmol/L) 3 weeks after the last cycle of chemotherapy. Patients with pre-existing renal insufficiency with creatinine over 150 μmol/L were excluded from the study. All patients provided informed written consent before enrolment. The relevant ethics committees in each participating center approved the study.

HE4 and CA125 serum levels were measured using ARCHITECT (Abbott, Chicago, IL, USA), an automated chemiluminescence immunoassay system. Venous blood samples were collected using the VACUETTE blood collection system (Greiner Bio-one Company, Rainbach im Mühlkreis, Austria). Blood was centrifuged for 10 min at 1700× *g*. Separated serum was immediately analyzed. Samples were analyzed in each participating center. If the Abbott ARCHITECT instrument was not available, it was possible to use any equivalent analyzer. Data on the sensitivity and specificity of HE4 for OC recurrence detection and the optimal cut-off level for a normal test result were not available when designing the study. Therefore, for this study, we evaluated two cut-off levels, ≤140 pmol/L, based on the level proposed by analyzers’ manufacturers (Abbott, Roche, Basel, Switzerland), and a cut-off level ≤70 pmol/L, suggested by Moore et al. [17] and used by Plotti et al. [18]. For patients with HE4 remission levels <70 pmol/L within three weeks after the end of first-line treatment, we set the HE4 cut-off level to perform a CT scan at >70 pmol/L set as HE4 normal. However, for those with HE4 remission levels between 71 and 140 pmol/L, the HE4 cut-off for requesting a CT scan was set at >140 pmol/L. The follow-up cut-off for CA125 was 35 IU/mL.

### 2.1. Study Procedures and Follow-Up

CA125 and HE4 were checked every 3–4 months in the first two years following primary treatment, then every six months until the fifth year post-primary-treatment. Bimanual gynecological examination together with transvaginal ultrasound were recommended at each visit. If there was an increase in one or both markers above the set cut-off or >20% increase compared to the previous result (already increased value above the set cut-off) at any of the follow-up time points, a CT examination of the abdomen and chest was performed within the three following weeks. The finding of new measurable lesions on CT, based on RECIST (Response Evaluation Criteria in Solid Tumours), was considered a first relapse, and this was considered a study endpoint for that patient. The study protocol did not require biopsy, diagnostic laparoscopy, or exploratory laparotomy to confirm recurrence. However, if the scan was normal, the patient continued to follow the biomarkers screening schedule. Tumor markers and a CT scan (abdomen and chest) were also performed at any time in case of clinical symptoms or any other imaging modality suggesting a possible ovarian cancer recurrence.

### 2.2. Power Analysis

Power analysis was based on the assumption that HE4 would be elevated five months prior to CA125 in case of a relapse. We estimated that 150 patients would be required to confirm this assumption if it existed. With a study length of 24 months, after recruitment was completed, 80 relapse events would have been expected. However, as fewer patients were enrolled in the recruitment period (N = 117), follow-up was extended to keep the number of events in accordance with the planned number to achieve a test power of 80%.

### 2.3. Statistical Analysis

Data were collected using software called CLADE-IS (v 23.04) (Clinical Data Warehousing Information System, Institute of Biostatistics and Analysis, Ltd., Brno, Czech Republic). Continuous variables were described using median and interquartile range (IQR) and categorical variables as absolute and relative frequencies. A receiver operating characteristic (ROC) analysis was applied to find cut-offs with the best sensitivity and specificity, and the results were assessed according to Youden’s J statistic, which maximizes the distance to the identity (diagonal) line. The Kaplan–Meier method was used to plot the time to relapse according to the baseline level of HE4. The difference between groups was expressed by the hazard ratio (HR) based on the Cox proportional hazards model. The univariate (crude) estimate was supplemented with the adjusted estimate from the multivariate model, where the covariates of age and grade were added. All tests were performed as two-sided at the 5% significance level. Analyses were performed in R software (v 4.3.2).

## 3. Results

Out of 120 screened patients, 117 met the inclusion criteria and were enrolled in the study.

Table 1 shows the population characteristics at baseline. Data from 616 post-baseline visits were recorded in the eCRFs. The median number of follow-up visits per patient was four. Of the 616 visits, 241 (39%) were unscheduled. The median follow-up was 13.7 months. Overall, 85 (73%) patients relapsed.

The median (IQR) HE4 level at the evidence of relapse (N = 85, 73% of patients) was 96 (69–204) pmol/L, whereas the median HE4 level in patients without relapse (N = 32, 27% of patients) was 45 (40–57) pmol/L at the last visit (*p* < 0.001). The median HE4 level at the preceding visit in the recurrence group was 59 (48–80) pmol/L. The median CA125 level at evidence of relapse was 76 (38–206) IU/mL, while at the last visit of patients without relapse, it was 11 (8–16) IU/mL. In the visit preceding relapse, the level was 16 (10–29) IU/mL. Thus, both HE4 and CA125 levels were significantly elevated in the visit preceding relapse (*p* < 0.001 and *p* = 0.001, respectively). However, only three (3.5%) patients with relapse had HE4 above 140 pmol/L at the preceding visit, and only thirteen (15.3%) patients with relapse and one (3.2%) without relapse had CA125 above 35 IU/mL at the preceding visit. In our study of 117 patients, we did not find a relationship between the currently used cut-off values for both markers and the early detection of recurrence.

Table 2 presents participants’ data at the last follow-up timepoint in the study for that participant (i.e., the visit in which relapse was confirmed (N = 85) or the last planned visit in patients without relapse (N = 32)).

The median absolute (relative) increase in HE4 from baseline (marker value when the patient achieved clinical remission) in patients with relapse was 52 (15–143) pmol/L (100% (33–230%)), whereas in patients without relapse, there was a median decrease of −4 (−11–1) pmol/L (−7% (−20–3%)).

The median absolute (relative) increase in CA125 from baseline (marker value when the patient achieved clinical remission) in patients with relapse was 68 (25–191) IU/mL (741% (245–1433%)), whereas in patients without relapse, there was no change 0 (−2–1) IU/mL (0% (−10–10%)).

**Figure 1:**
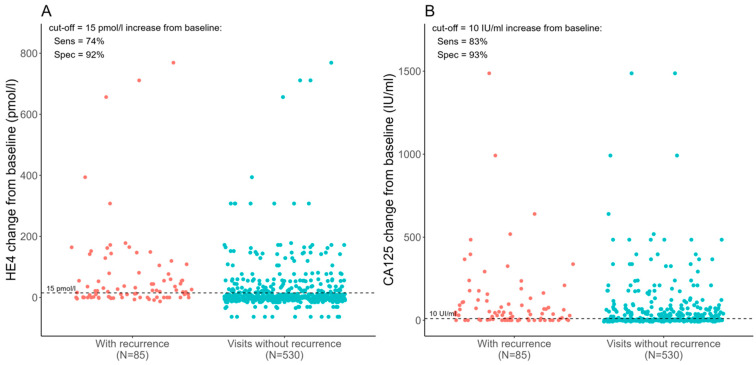
Changes of (**A**) HE4 and (**B**) CA125 tumor marker from baseline in patients with and without recurrence.

Our analysis indicated that an increase of 10 IU/mL in CA125 level from baseline (marker value when the patient achieved clinical remission) had a sensitivity of 83% and a specificity of 93% in detecting recurrence on average two months before CT scan. In the case of HE4, a 15 pmol/L absolute change from baseline had a sensitivity and specificity of 74% and 92%, respectively, in detecting recurrence 3.3 months before the CT scan (Figure 1).

The combination of markers CA125 and HE4 at defined absolute change levels of 10 IU/mL or more and 15 pmol/L or more has a sensitivity of 63% and a specificity of 97% in detecting recurrence.

**Figure 2:**
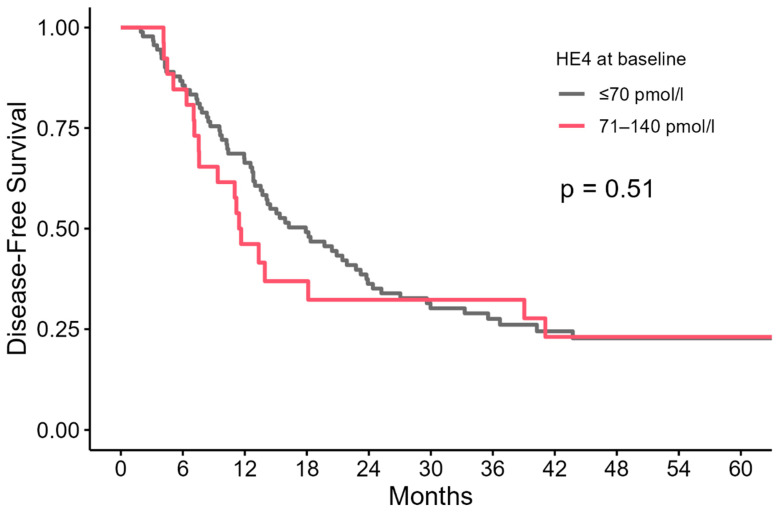
Relationship between baseline HE4 values and disease-free survival (DFS).

Our data did not show that a higher baseline HE4 value resulted in a significantly worse prognosis and earlier relapse (Figure 2, Table 3).

## 4. Discussion

The roles of CA125 and HE4 in the diagnosis and monitoring of ovarian cancer differ significantly and have been the subject of extensive debate within the medical community for decades. Nevertheless, their clinical utility remains constrained by several factors, including limitations in sensitivity, specificity, and their impact on overall survival (OS). Our study explored the potential for detecting disease recurrence during follow-up after first-line therapy by comparing marker elevation with concurrent imaging modalities to assess their temporal differences. Additionally, the study evaluated the accuracy of current cut-off values for the early detection of relapse and examined the necessity of dynamic monitoring.

In a prospective controlled study by Plotti et al., the sensitivity and specificity of HE4, either alone or in combination with other markers (CA125, CA72-4), were superior to those of CA125 alone in diagnosing ovarian cancer relapse. Combining CA125 and HE4 at a cut-off of 70 pmol/L achieved a sensitivity of 76% and a specificity of 100% for detecting ovarian cancer recurrence, compared to a sensitivity of 35% and a specificity of 59% when relying solely on CA125 [18].

In the study by Lakshmanan, the sensitivity of serum HE4 in detecting recurrence was 85.3% (95% CI: 76.9–91.5%), and specificity was 91.5% (95% CI: 79.6–97.6%). The positive predictive value for serum HE4 was 95.6% (95% CI: 89.5–98.2%), and negative predictive value was 74.1% (95% CI: 80.8–92.1%). The mean lead time of serum HE4 over serum CA125 in detecting recurrence was found to be 2.76 months, and median lead time was 3 months [19].

Interestingly, in our study, the established cut-off values for HE4 (140 pmoL/L in postmenopausal women) and CA125 (35 IU/mL) did not demonstrate significance in the early detection of relapse. In the recurrence group, CA125 levels exceeded the normal threshold (35 IU/mL) in 64 patients (77%), which gave a sensitivity of 77% with a specificity of 94%, while HE4 levels surpassed 140 pmol/l in 31 patients (37%), which gave a sensitivity of only 37% with a specificity of 100%.

Based on consecutive measurements of tumor marker levels, standard cut-off values of HE4 and CA125 did not demonstrate reliable sensitivity and specificity for early relapse detection. However, focusing on the dynamic changes in these markers, even when they remain within normal ranges, revealed their predictive value for relapse. An increase of 10 IU/mL in CA125 (sensitivity 83%, specificity 93%) and 15 pmoL/L in HE4 (sensitivity 74%, specificity 92%), with a combined sensitivity of 63% and specificity of 97%, strongly indicates the likelihood of recurrence at these levels. Rustin et al. found a critical value of two or more times the normal upper limit for the CA125 marker [20]. The HE4 marker was not investigated in this study.

Routine follow-up using CT scans did not significantly enhance the overall detection of early relapses in ovarian carcinoma. The accuracy of CT imaging for detecting recurrent disease varies widely, ranging from 38% to 88% [21]. This variability is primarily due to its low sensitivity in detecting small tumors and insufficient resolution to distinguish between postoperative changes and pathological findings. Our study demonstrated that consecutive monitoring of tumor markers CA125 and HE4 every 3–4 months can provide an early warning of an impending recurrence. Routine CT scans are not able to detect the disease as accurately or as early as the tumor markers CA125 and HE4. However, the disease may become visible on follow-up scans after several months. Our results open the door to a new prospective study to evaluate the sensitivity and specificity of advanced imaging modalities, such as PET/CT or PET/MRI, in the early detection of ovarian carcinoma. The suggested imaging study would focus on cases where tumor markers begin to rise but still remain within normal reference ranges.

A study by Rustin et al. showed that CA125 levels rise approximately 3 to 5 months before clinical or radiological evidence of recurrence. Despite this early detection, the initiation of treatment based solely on CA125 elevation did not improve overall survival in Rustin’s study [20]. This could be the main criticism of our study, i.e., that our published data, considering the Rustin study cited above, do not provide a solution or rational application of the results obtained. In light of new treatment modalities and approaches, this impact needs to be verified.

HE4 has been demonstrated as an earlier indicator of ovarian cancer (OC) recurrence compared to CA125, with a lead time of 5 to 8 months [22]. However, only a limited number of studies have evaluated its use in OC follow-up to date [18,22,23,24,25,26,27], and these studies involved relatively small patient cohorts (8–73 patients). Our study confirmed that HE4 serves as an earlier indicator of impending relapse detectable on CT, compared to the traditional marker CA125. Our findings show that elevation of the CA125 marker precedes detection of recurrence on CT by two months and elevation of the HE4 marker by three months.

Early detection of recurrence could allow patients to benefit from timely second-line chemotherapy or the opportunity for complete surgical resection of the recurrent disease, thereby improving their overall prognosis. Considering the new targeted therapeutical possibilities offered based on genetic testing, somatic mutation analysis, novel maintenance therapy, and advancements in second-line treatment, the results of the study by Rustin et al. on OS need to be questioned. Morris and Monk [28] also criticized Rustin and colleagues’ [20] trial for its interpretation of results, noting that it did not account for the issue of platinum sensitivity. The platinum-free interval was imbalanced, because some patients began treatment early while others experienced delays. Additionally, contemporary therapies, such as bevacizumab and pegylated liposomal doxorubicin plus carboplatin, were not available to most trial participants. Furthermore, it is crucial to highlight that the results of PARP inhibitor (poly-ADP ribose polymerase inhibitor) maintenance therapy, which are relevant for today’s comparisons, were not included.

Intensive follow-up based on consecutive monitoring of tumor markers CA125 and HE4, regardless of established cut-off values, and responding to “critical” elevations even within the normal range, may significantly improve overall survival through the early initiation of targeted second-line therapy when recurrence is confirmed on CT. This hypothesis requires confirmation in a new prospective study.

We acknowledge several limitations in our study. First, patients were provided with clinically indicated standard follow-up, which may not have included offering advanced or experimental treatments. Second, our protocol required extensive diagnostic imaging after tumor marker elevation (including CT scans of the chest and abdomen) and biomarker analysis (CA125, HE4) prior to inclusion and at each subsequent visit. While essential for monitoring disease status, this rigorous assessment could have imposed physical and logistical burdens on participants. The intensive follow-up, particularly with frequent blood sampling for tumor markers HE4 and CA125, may have contributed to patient stress and anxiety, especially in cases where rising marker levels suggested an early indication of recurrence without accompanying clinical or radiographic confirmation. An increase in tumor markers without corresponding clinical or CT evidence of recurrence presented a challenging scenario. At the same time, it did not justify the initiation of chemotherapy, and it could have significantly impacted patients’ psychological well-being and quality of life due to concerns over potential disease progression.

Conversely, our study also had several strengths. The intensive follow-up regimen may have provided some patients a heightened sense of security, as closer monitoring allowed for the early detection of recurrence, theoretically enabling timely intervention. Moreover, participation in the study contributed valuable data to the medical community, potentially benefiting future patients through improved understanding and management of ovarian cancer recurrence.

## 5. Conclusions

Our study highlights that tracking dynamic changes in CA125 and HE4 levels is essential for the effective follow-up of ovarian cancer patients. Absolute marker values are not reliable for early relapse detection; instead, monitoring trends and increases from baseline offers a more accurate prediction of recurrence. Importantly, combining CA125 and HE4 does not improve prediction accuracy compared to using either marker alone. Regular, periodic monitoring of CA125 and HE4 remains crucial for early detection of relapse. Further research is required to refine follow-up strategies and enhance outcomes for ovarian cancer patients.

## Figures and Tables

**Table 1:** The population characteristics at baseline.

	Overall, N = 117 ^1^	Recurrence, N = 85 ^1^	No Recurrence, N = 32 ^1^	*p*-Value ^2^
Age	62 (51–68)	63 (52–69)	59 (46–66)	0.176
Pre-treatment HE4 [pmol/L]	291 (125–680)	400 (176–798)	155 (97–345)	0.001
Pre-treatment CA125 [IU/mL]	418 (143–1000)	472 (207–1031)	145 (67–522)	0.001
3 weeks post-treatment mean HE4 [pmol/L]	53 (42–65)	53 (42–65)	53 (41–63)	0.959
3 weeks post-treatment mean CA125 [IU/L]	10 (8–16)	10 (7–17)	11 (9–15)	0.668
End of first-line chemotherapy—CT thorax residual disease				>0.999
No lesion	117 (100.0%)	85 (100.0%)	32 (100.0%)	
Lesion according RECIST criteria	0 (0.0%)	0 (0.0%)	0 (0.0%)	
End of first-line chemotherapy—CT abdomen residual disease				>0.999
No lesion	117 (100.0%)	85 (100.0%)	32 (100.0%)	
Lesion according RECIST criteria	0 (0.0%)	0 (0.0%)	0 (0.0%)	
CR after the first-line treatment according to CT abdomen/thorax; HE4 and CA125	117 (100.0%)	85 (100.0%)	32 (100.0%)	
Biological treatment				>0.999
No	102 (87.2%)	73 (85.9%)	29 (90.6%)	
Bevacizumab	13 (11.1%)	10 (11.8%)	3 (9.4%)	
Olaparib	1 (0.9%)	1 (1.2%)	0 (0.0%)	
Other	1 (0.9%)	1 (1.2%)	0 (0.0%)	
Chemotherapy				
Paclitaxel, Carboplatin	116 (99.4%)	84 (98.2%)	32 (100.0%)	
Unknown	1 (0.9%)	1 (1.2%)	0 (0.0%)	
Histology				0.910
Ovarian cancer	96 (82.1%)	70 (82.4%)	26 (81.2%)	
Fallopian tube cancer	16 (13.7%)	11 (12.9%)	5 (15.6%)	
Peritoneal cancer	5 (4.3%)	4 (4.7%)	1 (3.1%)	
Histotype				0.382
High-grade serous	104 (88.9%)	77 (90.6%)	27 (84.4%)	
Low-grade serous	5 (4.3%)	2 (2.4%)	3 (9.4%)	
Endometroid	3 (2.6%)	2 (2.4%)	1 (3.1%)	
Clear cell	5 (4.3%)	4 (4.7%)	1 (3.1%)	
Undifferentiated	0 (0.0%)	0 (0.0%)	0 (0.0%)	
Grade				0.117
Grade 1	4 (3.4%)	2 (2.4%)	2 (6.2%)	
Grade 2	3 (2.6%)	1 (1.2%)	2 (6.2%)	
Grade 3	100 (85.5%)	76 (89.4%)	24 (75.0%)	
Unknown	10 (8.5%)	6 (7.1%)	4 (12.5%)	
FIGO classification				0.298
IIIA1	9 (7.7%)	4 (4.7%)	5 (15.6%)	
IIIA2	5 (4.3%)	4 (4.7%)	1 (3.1%)	
IIIB	14 (12.0%)	11 (12.9%)	3 (9.4%)	
IIIC	74 (63.2%)	54 (63.5%)	20 (62.5%)	
IVA	6 (5.1%)	6 (7.1%)	0 (0.0%)	
IVB	9 (7.7%)	6 (7.1%)	3 (9.4%)	
Date of enrollment	30 June 2016–9 December 2020	30 June 2016–12 December 2020	10 October 2016–21 December 2020	0.883
HE4 group				0.956
≤70	91 (77.8%)	66 (77.6%)	25 (78.1%)	
71–140	26 (22.2%)	19 (22.4%)	7 (21.9%)	
Samples analyzed on				0.199
Abbott ARCHITECT	74 (63.2%)	57 (67.1%)	17 (53.1%)	
Roche	43 (36.8%)	28 (32.9%)	15 (46.9%)	
Other	0 (0.0%)	0 (0.0%)	0 (0.0%)	
Number of visits	5.0 (3.0–9.0)	4.0 (3.0–6.0)	11.0 (8.8–12.5)	<0.001
Length of follow-up (months)	14 (8–33)	12 (7–18)	45 (32–61)	<0.001
PI (postmenopausal)	2.16 (0.73–3.67)	2.78 (1.23–3.87)	0.82 (−0.01–2.06)	<0.001
ROMA (%)	90 (68–98)	94 (77–98)	69 (50–89)	<0.001

^1^ Median (IQR); range; n (%). ^2^ Mann–Whitney *U* test; Fisher’s exact test; Pearson’s Chi-squared test.

**Table 2:** Changes in markers during follow-up.

	Recurrence N = 85 ^1^	No Recurrence N = 32 ^1^	*p*-Value ^2^
LAST VISIT			
HE4 at last FUP visit (pmol/L)	96 (69–204)	45 (40–57)	**<0.001**
HE4 over 140 pmol/L	31 (37.3%)	0 (0.0%)	**<0.001**
CA125 at last FUP visit (IU/mL)	76 (38–206)	11 (8–16)	**<0.001**
CA125 over 35 IU/mL	64 (77.1%)	2 (6.5%)	**<0.001**
SECOND-TO-LAST VISIT			
Number of days before the last visit	105 (91–133)	181 (132–199)	**<0.001**
HE4 at second-to-last visit (pmol/L)	59 (48–80)	44 (38–56)	**<0.001**
HE4 over 140 pmol/L	3 (3.5%)	0 (0.0%)	0.563
CA125 at second-to-last visit (IU/mL)	16 (10–29)	11 (8–14)	**0.001**
CA125 over 35 IU/mL	13 (15.3%)	1 (3.2%)	0.108
CHANGE FROM BASELINE			
HE4 at baseline (pmol/L)	53 (42–65)	53 (41–65)	0.915
HE4 change from baseline (pmol/L)	52 (15–143)	−4 (−11–1)	**<0.001**
HE4 change from baseline (%)	100 (33–230)	−7 (−20–3)	**<0.001**
CA125 at baseline (IU/mL)	10 (7–17)	11 (9–16)	0.547
CA125 change from baseline (IU/mL)	68 (25–191)	0 (−2–1)	**<0.001**
CA125 change from baseline (%)	741 (245–1433)	0 (−10–10)	**<0.001**
TYPE OF RECURRENCE			
Local	34 (40.0%)		
Regional	50 (58.8%)		
Distant	64 (75.3%)		

^1^ Median (IQR); n (%). ^2^ Mann–Whitney *U* test; Pearson’s Chi-squared test; Fisher’s exact test. Bold numbers indicate statistical significance.

**Table 3:** HE4 value at baseline and related prognosis and relapse.

	Median DFS (Months)		1-Year Survival (95% CI)		3-Year Survival (95% CI)	5-Year Survival (95% CI)
HE4 at baseline (pmol/L)						
≤70	18		0.66 (0.57–0.77)		0.28 (0.19–0.39)	0.23 (0.15–0.34)
71–140	12		0.46 (0.30–0.70)		0.32 (0.18–0.58)	0.23 (0.11–0.49)
			**Crude HR Estimate**		**Adjusted ^1^ HR Estimate**	
	**N**	**Event N**	**HR (95% CI)**	***p*** **-Value**	**HR (95% CI)**	***p*** **-Value**
HE4 at baseline (pmol/L)						
≤70	91	66	-		-	
71–140	26	19	1.19 (0.71–1.98)	0.514	1.11 (0.65–1.90)	0.710

DFS = disease-free survival, HR = hazard ratio, CI = confidence interval. ^1^ Adjusted for age and grade.

## Data Availability

The datasets generated and/or analyzed during the current study are available from the corresponding author on reasonable request.
